# Defensive Traits in Young Pine Trees Cluster into Two Divergent Syndromes Related to Early Growth Rate

**DOI:** 10.1371/journal.pone.0152537

**Published:** 2016-03-30

**Authors:** Xoaquín Moreira, Luis Sampedro, Rafael Zas, Ian S. Pearse

**Affiliations:** 1 Misión Biológica de Galicia (MBG-CSIC), Apdo. 28, 36080 Pontevedra, Galicia, Spain; 2 Illinois Natural History Survey, 1816 South Oak Street, Champaign, Illinois, 61820, United States of America; Umeå Plant Science Centre, Umeå University, SWEDEN

## Abstract

The combination of defensive traits leads to the evolution of ‘plant defense syndromes’ which should provide better protection against herbivores than individual traits on their own. Defense syndromes can be generally driven by plant phylogeny and/or biotic and abiotic factors. However, we lack a solid understanding of (i) the relative importance of shared evolution vs. convergence due to similar ecological conditions and (ii) the role of induced defense strategies in shaping defense syndromes. We investigate the relative roles of evolutionary and ecological factors shaping the deployment of pine defense syndromes including multiple constitutive and induced chemical defense traits. We performed a greenhouse experiment with seedlings of eighteen species of Pinaceae family, and measured plant growth rate, constitutive chemical defenses and their inducibility. Plant growth rate, but not phylogenetic relatedness, determined the deployment of two divergent syndromes. Slow-growing pine species living in harsh environments where tissue replacement is costly allocated more to constitutive defenses (energetically more costly to produce than induced). In contrast, fast-growing species living in resource-rich habitats had greater inducibility of their defenses, consistent with the theory of constitutive-induced defense trade-offs. This study contributes to a better understanding of evolutionary and ecological factors driving the deployment of defense syndromes.

## Introduction

Historically, most plant defense theories predict the occurrence of trade-offs between different types of anti-herbivore defenses [1,2]. However, recent studies have challenged this view and argue that, in order to cope with a wide range of herbivore species, plants optimize an arsenal of diverse traits in concert, including plant chemical and physical defenses, indirect defenses, plant nutritional quality and tolerance mechanisms [3–10]. Besides, the phenotypic combination of multiple defenses is usually not random. In a seminal paper, Agrawal & Fishbein [3] extended the concept of animal behavioral syndromes [11] to plants and proposed that defense syndromes, defined as correlated suites of defensive traits covarying at the species or population level across environments, may be prevalent in nature.

In general, plant defense syndromes might be driven by shared evolution or homoplasious solutions to biotic and abiotic environmental challenges [3]. For instance, phylogenetically related plant species that share a common assemblage of herbivore species frequently possess a suite of similar chemical defensive traits or strategies [12–14]. Alternatively, unrelated plant species growing under relatively similar ecological conditions and herbivore pressures might evolve convergent suites of chemical defensive traits [15,16]. Although the generality of plant defense syndromes have been described for several plant families (e.g. [3,5,8,10,17–20]), (i) we often ignore the relative importance of shared evolution vs. convergence (due to similar ecological conditions) in shaping plant defense syndromes, and (ii) induced chemical defensive strategies has been rarely incorporated into the framework of plant defense syndromes.

Plant chemical defenses can be broadly classified as constitutive (always expressed) or induced (expressed in response to perceived pathogen- or herbivore-damage) [21,22]. The benefits and costs of both defensive strategies vary depending on both the biotic and abiotic context in which plants are embedded [23]. For example, environments with low herbivore pressure should select for induced chemical defenses over constitutive chemical defenses to reduce costs associated with defense production; conversely, environments with high and constant herbivore pressure should select for constitutive chemical defenses [24]. Based on this, induced defenses are thought to have evolved as a cost-saving strategy as they are only produced when necessary [25,26]. Consequently, and based upon the Resource Availability Hypothesis, unfavorable environments that favor slow-growing strategies should select also for increased allocation to constitutive chemical defenses (energetically more costly than induced) as plants cannot easily replace damaged tissues that would represent a large portion of their cumulative growth [16,27]. Because secondary metabolism is costly for plants, and both strategies (constitutive and induced) are not likely to be maximized simultaneously [28,29], well constitutively-defended species growing in more stressful environments are expected to gain little from evolving induced responses.

Induced chemical defenses are energetically more efficient but may not be activated quickly enough. A plant might remain vulnerable for a period of time until induced defenses are deployed. The delay time between attack and the activation of the induced chemical defenses might be in some cases very long to be effective [30]. Therefore, as the efficiency of constitutive and induced defensive strategies are context dependent and not favored simultaneously, divergent defensive syndromes could be expected. Assessing the strength and direction of relationships between growth and both constitutive and induced defensive strategies would help to determine whether chemical defensive traits act in concert or antagonistically.

Two recent studies by our research group evidenced a trade-off between induced and constitutive expression of chemical defenses across pines species, and that the expression of multiple individual defensive traits is constrained by phylogeny, climate and geographic clines, likely determining growing strategies [28,31]. In particular, we found a strong phylogenetic signal in the constitutive concentration of phenolics in needles and of resin produced in the stem [31]. We also found that the constitutive concentration of resin in the stem traded off with the induced concentration of resin and growth rate, such that slow growing species invested more in constitutive vs. induced levels of resin production, and vice versa [28]. Here using the same dataset we go a step forward and investigate the relative roles of evolutionary (phylogeny) and ecological factors (constraints on the allocation to growth, constitutive and induced defenses) shaping the deployment of pine defense syndromes, including multiple constitutive and induced chemical defensive traits. We specifically addressed three questions: (i) Are defensive phenotypes of pine species clustered into defensive syndromes that simultaneously include both constitutive and induced defensive traits? (ii) Are defensive syndromes driven by shared evolutionary history, or do unrelated plants converge on the same set of syndromes? And (iii) do plant growth rate shape pine defense syndromes? To address these questions, we used data from a previous greenhouse experiment with juveniles of eighteen species of Pinaceae family trees distributed across a broad range of environmental conditions in both Nearctic and Palearctic ranges [28,31]. We measured plant growth rate and the constitutive investment in the two most important chemical defenses in conifers (phenolics and oleoresin) in the needles and the stem [32], as well as the inducibility (i.e., the ability to increase defenses in response to external challenges) of those defensive traits as driven by the jasmonic acid (JA) and the salicylic acid (SA) signaling pathways [33]. Overall, the current study builds towards a better understanding of evolutionary and ecological factors driving the deployment of defense syndromes in one of the most commercially important tree genus throughout the world.

## Material and Methods

### Ethics Statement

The research did not involve manipulations of humans or animals. No specific permissions were required for our greenhouse work. The study did not involve endangered or protected species.

### Natural history

This study included 18 species in the family Pinaceae that cover large forested areas in the Northern Hemisphere. Seventeen of these species belong to the genus *Pinus* (*Pinus canariensis*, *P*. *halepensis*, *P*. *nigra* subsp. *salzmannii* var. *corsicana*, *P*. *nigra* subsp. *salzmannii var*. *hispanica*, *P*. *pinea*, *P*. *pinaster* and *P*. *sylvestris* have a Paleartic range; while *P*. *banksiana*, *P*. *contorta*, *P*. *coulteri*, *P*. *muricata*, *P*. *patula*, *P*. *ponderosa*, *P*. *radiata*, *P*. *roxburghii*, *P*. *sabiniana*, *and P*. *taeda* have a Neartic range); and one to the genus *Pseudotsuga* (*Pseudotsuga menziesii*, Neartic range) [28,31]. We chose *P*. *menziesii* for our experiment because this species has similar defensive mechanisms than other pine species [31] and coexists in mixed forests with most of the pine species in western North America (e.g. *P*. *contorta*, *P*. *ponderosa*, *P*. *coulterii*) and host the same herbivores [34]. Species of Pinaceae family provide an excellent system to address our questions because they are broadly distributed in a very wide range of habitats and are attacked by a wide range of different herbivores (more than 350 species), many of which co-exist on the same host plant and likely favor the concerted expression of multiple defensive mechanisms and strategies [34,35].

Pine defense is mainly based on the constitutive production and inducibility of carbon-based secondary compounds (resin and phenolic compounds) that are present in all pine tissues at high concentrations and represent 1–10% of dry mass in different tissues, and about 2–5% of total biomass at the juvenile stage [28,32,36–38]. Both constitutive and induced resin and phenolic compounds provide resistance against a diverse array of herbivores and pathogens [28,32,39].

### Experimental design

The greenhouse experiment included two main factors, conifer species with the 18 species and treatments of plant defense induction, with three levels, namely: an untreated control for studying the constitutive allocation to chemical defenses, a jasmonic acid (JA)-induced treatment, and a salicylic acid (SA)-induced treatment (hereafter “treatments”). JA is a plant hormone essential for damage signaling and resistance against chewing herbivores and necrotrophic pathogens. In contrast, SA is a plant hormone involved in resistance against biotrophic pathogens and phloem-sucking insects. The SA and JA signaling pathways have been described often as antagonistic [33]. The experiment followed a randomized split-plot design replicated in 10 blocks, with treatments as the whole-plot factor and pine species as the split-plot factor. Due to reduced availability of plant material for some pine species, we only included 15 species in the SA treatment. In total, the experiment included 510 juveniles, corresponding to 10 replicate blocks with 18 species subjected to control and JA treatments, plus 10 replicate blocks with 15 of those species subjected to SA treatment.

### Plant growth, greenhouse conditions, and treatments

In October 2008, we individually sowed pine seeds in 2-L pots filled with a mixture of perlite and peat (1:1 v:v), fertilized them with 12 g of a slow-release fertilizer (N:P:K 15:15:15, Multicote®, Haifa Chemicals Ltd., Israel), and covered them with a 1–2 cm layer of sterilized sand. We purchased seeds from commercial sources (Intersemillas S.A., Spain; Les Semences du Pay, France). As far was possible we got seeds from the mid-range of each species distribution area. To avoid interference from pathogens, we treated seeds with a fungicide before sowing (Fernide®, Syngenta Agro, Madrid, Spain). We grew all the plants in a research grade glasshouse with controlled temperature (10°C minimum temperature at night; 25°C maximum temperature during daytime) and light (minimum 12 h per day), and automatic daily sub-irrigation [28].

Thirteen months after emergence, we applied treatments: i) JA-treated plants were sprayed with a solution of 40 mM methyl jasmonate (MJ; Sigma-Aldrich, #39270–7, St. Louis, MO, USA) in deionized water with ethanol 2.5% (v:v); ii) SA-treated plants were sprayed with a solution of 5 mM benzo-(1,2,3)-thiadiazole-7-carbothioic acid S-methyl ester (BTH, Syngenta Bion® 50 WG) in deionized water with ethanol 2.5% (v:v); (iii) control-treatment plants were sprayed with the carrier solution, i.e. deionized water with ethanol 2.5% (v:v). We sprayed the treatments evenly over the foliage with a handheld sprayer until runoff occurred. MJ and BTH are synthetic analogues of jasmonic acid and salicylic acid, respectively. We determined the appropriate concentrations of MJ and BTH according to previous studies with pine juveniles grown under similar conditions (e.g. [36,37,40]). To avoid cross-contamination, we applied treatments in separate greenhouse chambers and plants remained in those separate spaces for 48 h to dry.

### Sampling, measurements, and chemical analyses

Two weeks after treatment application, we harvested all plants and transported them to the laboratory in ice coolers and immediately sampled them for further chemical analyses. Shortly after harvesting, we collected a fresh 5-cm-long segment of stem and a sample of needles (ca. 5 g. fresh weight) from the lowest part of the stem of each plant, and immediately froze these samples at -30°C for analysis of non-volatile resin content and total phenolics. We measured the concentration of total phenolics and non-volatile resin in needles and stems of all experimental plants.

We estimated gravimetrically the concentration of non-volatile resin in the stem (phloem + xylem) and needles as in Moreira et al. [37]. Specifically, we transferred plant material (ca. 5 g fresh weight) into preweighed test tubes, extracted oleoresin with hexane (15 min sonication and then 24 h at room temperature), quantitatively transferred the extract into preweighed test tubes, and repeated the entire extraction step again. The solvent in the tubes was evaporated to dryness under a fume hood. We determined the mass of the non-volatile resin residue at the nearest 0.0001 g and expressed the concentration as mg of non-volatile resin g^-1^ stem d.w. Gravimetric determination of non-volatile resin was highly correlated with the concentration of the diterpenoid fraction (r = 0.921; *P*<0.001) as quantified by gas chromatography [36]. We extracted total phenolics in the stem and needles and analyzed them as described by Moreira et al. [37]. Specifically, we finely ground ca. 2 g of oven-dried plant material (45°C to constant weight) in liquid N and extracted ca. 300 mg with aqueous methanol (1:1 vol:vol) after sonication (15 min). We determined colorimetrically total phenolics in the extract by the Folin-Ciocalteu method in a Biorad 650 microplate reader (Bio-Rad Laboratories Inc., Philadelphia, PA, USA) at 740 nm, using tannic acid as standard. We expressed the concentrations based on dry weights (d.w.). Previous studies have demonstrated that these analytical measures are good proxies of quantitative allocation to defensive chemistry and strongly correlate with effective resistance against herbivores in young pine trees (see supplementary material in Moreira et al. [28]). In total, we measured 12 defensive traits corresponding to two chemicals (resin and phenolics) evaluated in two tissues (stem and needles) under constitutive, JA induced and SA induced conditions. For each species, inducibility of a given trait was estimated as the difference in mean phenotype values between induced plants and control plants.

### Statistical analyses

The analyses of concentration of constitutive chemical defenses and their inducibility in stems and needles were carried out by fitting a mixed model (PROC MIXED in SAS 9.2, Cary, NC) with the main effects of treatments (T), pine species (SP), and T × SP interaction treated as fixed factors, and the block (B) and B × T interaction as random factors in order to test the whole-plot factor (T) with the appropriate error term [41]. From this statistical model we obtained least square means ± standard error of the mean (s.e.m.) as descriptive statistics for each species, under both control and each induced treatment, and for both stem and needle tissues [28]. These least square means were used for the estimation of the inducibility traits as described before.

#### Pine defense syndromes

Firstly, we constructed a trait distance matrix based on phenotypic functional defensive traits of pine species. We defined patterns of trait similarity among species using hierarchical cluster analysis to create a defense phenogram. Clustering was accomplished using Ward’s method [42,43]. We further described the two main clusters (hereafter Cluster A and B) using linear discriminant analysis (lda function in MASS package in R), and we display lda loading values for each defensive trait [3]. Significance tests of how a particular trait differs between the two observed clusters were calculated from T-tests [3]. Secondly, we used a Mantel test to compare the phylogenetic tree for the genus *Pinus* published by Eckert & Hall [44] with the phenogram based on defensive traits of pine species. A significant difference from this test indicates that phylogeny significantly explains patterns of similarity in defense across pine species [3]. Finally, in order to determine whether pine defense syndromes were closely related to plant growth, we evaluated the effect of defensive clustering (Cluster A vs. Cluster B) on pine growth rate by using phylogenetic generalized least-squares models (PGLS; [45]).

## Results

**Fig 1:**
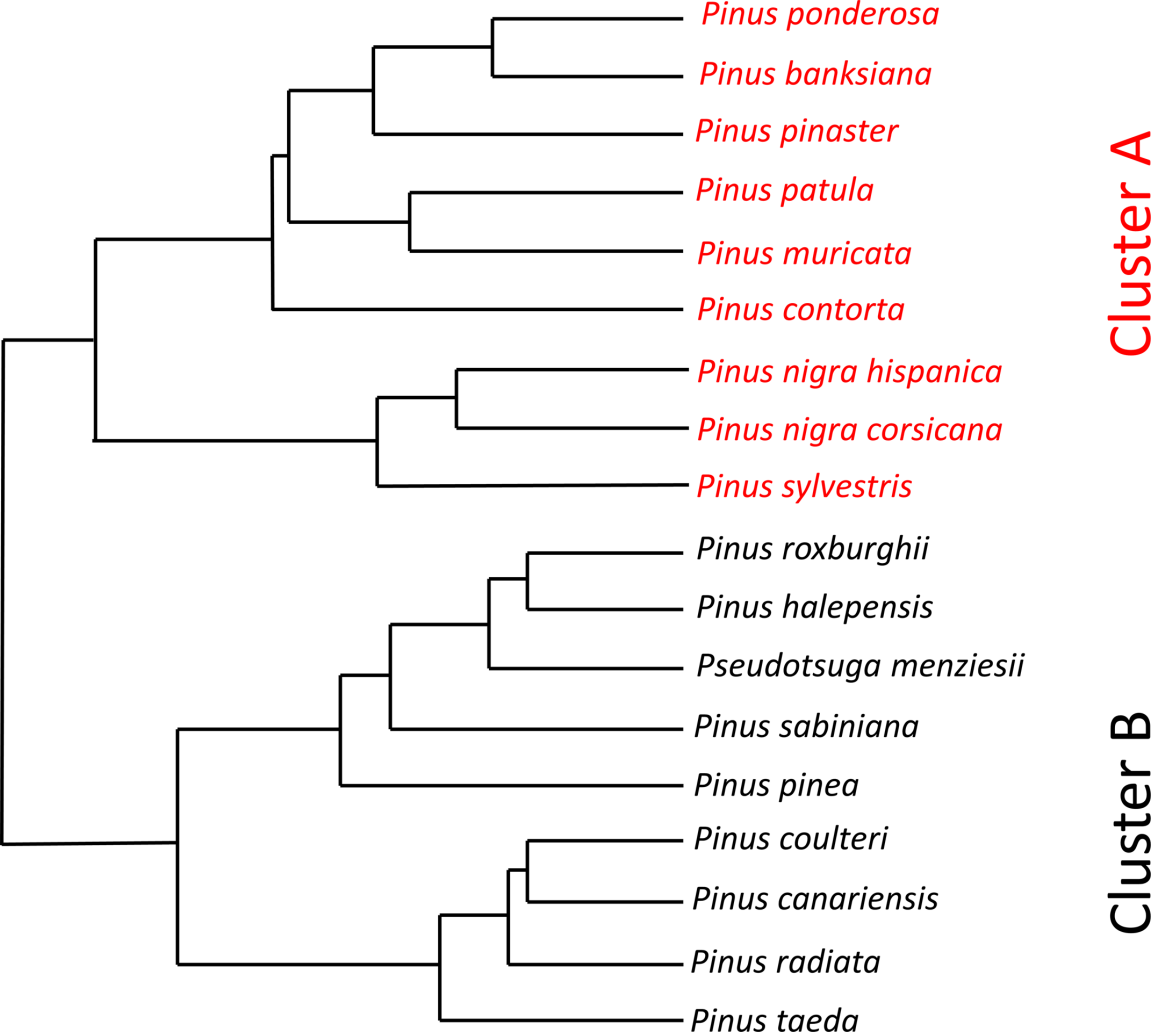
Defensive phenogram showing similarity among 18 conifer species of four constitutive defensive traits, four defensive traits induced by the jasmonic acid signaling pathway and four defensive traits induced by the salicylic acid pathway. Closely clustered species show similar integrated defensive phenotypes and form two defense syndromes (Cluster A in red font and Cluster B in black font).

Firstly, by using a hierarchical cluster analysis of constitutive defensive traits and their induciblity by JA and SA we identified two distinct clusters (i.e. two defense syndromes, Fig 1, Table 1). The first syndrome (Cluster A in Fig 1) represented conifer species (n = 9) with high levels of constitutive resin in the needles and stem (Table 1). The second syndrome (Cluster B in Fig 1) represented conifer species (n = 9) with high inducibility of resin and phenolics in the needles by both JA and SA pathways (Table 1).

**Table 1:** Contribution of the studied pine constitutive defensive traits and their inducibility to the defensive clustering of pine species. Trait values (least square mean ± SE) for the two defensive clusters (syndromes) and coefficients for linear discriminant analyses (LDA scaling) are shown. Significant (*P* < 0.05) and marginal (*P* < 0.10) differences between clusters are typed in bold.

Defensive trait	LDA scaling	Cluster A (n = 9)	Cluster B (n = 9)	*P*
Constitutive stem resin	106.81	39.80 ± 6.82	17.50 ± 1.21	**0.003**
Constitutive needle resin	-241.08	22.40 ± 2.25	17.30 ± 2.08	**0.097**
Constitutive stem phenolics	117.64	11.44 ± 0.56	11.95 ± 1.40	0.721
Constitutive needle phenolics	-182.87	23.95 ± 4.45	22.97 ± 1.65	0.829
JA-induced stem resin	65.43	1.64 ± 2.35	1.82 ± 1.16	0.944
JA-induced needle resin	128.33	-2.74 ± 2.08	5.59 ± 1.81	**0.007**
JA-induced stem phenolics	-66.05	-0.73 ± 1.10	0.42 ± 0.59	0.346
JA-induced needle phenolics	167.13	-5.15 ± 2.25	7.35 ± 1.75	**<0.001**
SA-induced stem resin	19.42	-4.47 ± 2.26	-0.45 ± 0.90	0.144
SA-induced needle resin	112.67	-4.22 ± 1.05	2.16 ± 2.23	**0.024**
SA-induced stem phenolics	-96.92	0.61 ± 1.68	-2.70 ± 1.52	0.183
SA-induced needle phenolics	NC^1^	-4.34 ± 1.70	3.15 ± 2.50	**0.030**

^1^NC = not calculated due to high collinearity with other traits

**Fig 2:**
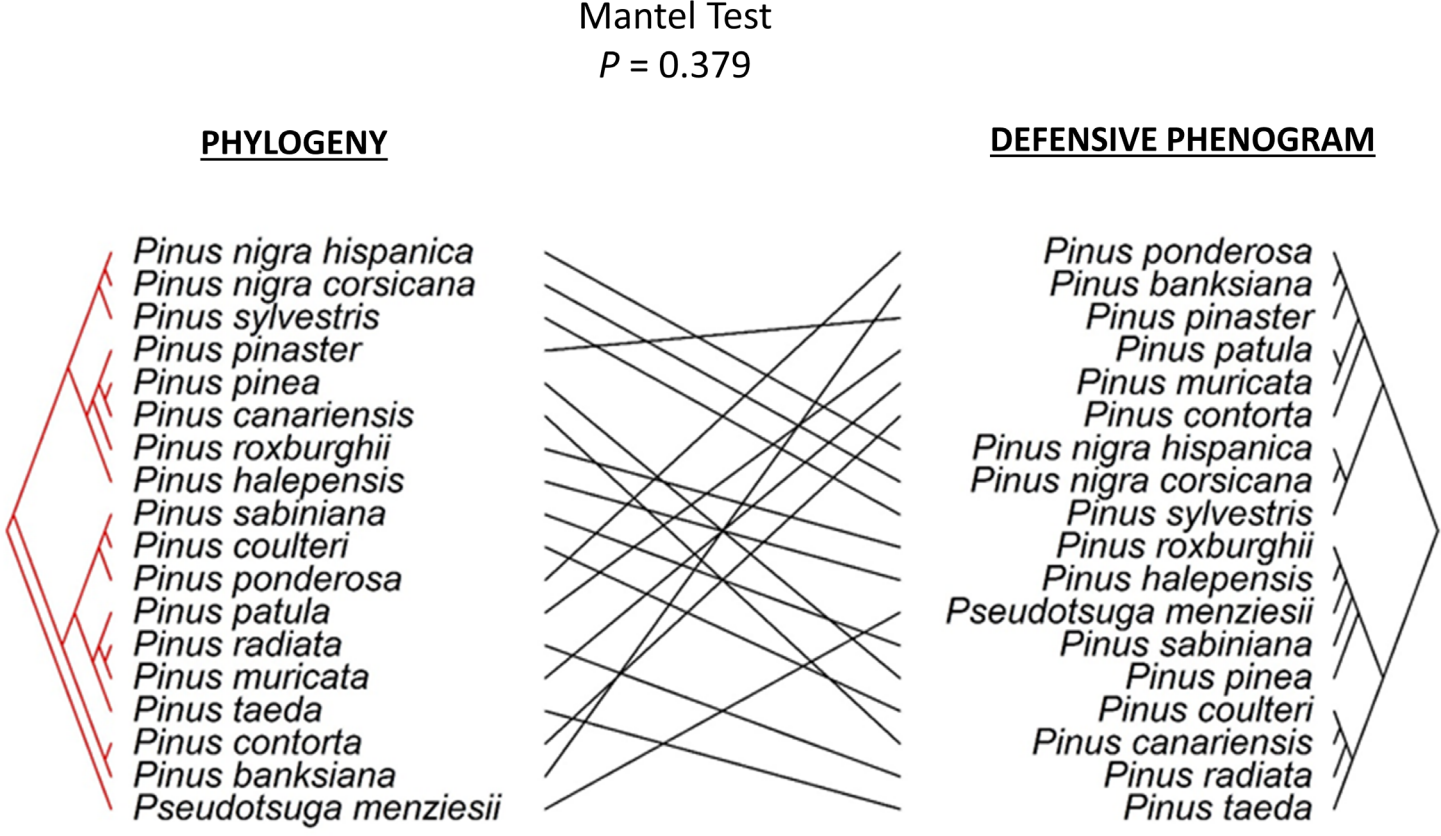
Scheme showing the lack of congruence between the molecular phylogeny of pine trees based on molecular markers by Eckert & Hall [44] and the defense phenogram constructed from phenotypic functional defensive traits in this study (Mantel Test, *P* = 0.379).

Secondly, a Mantel test showed that the proximity among species observed in the cluster analysis of phenotypic functional defensive traits was incongruent with the phylogenetic relationships estimated from the molecular analysis by Eckert & Hall [44] (Fig 2).

**Fig 3:**
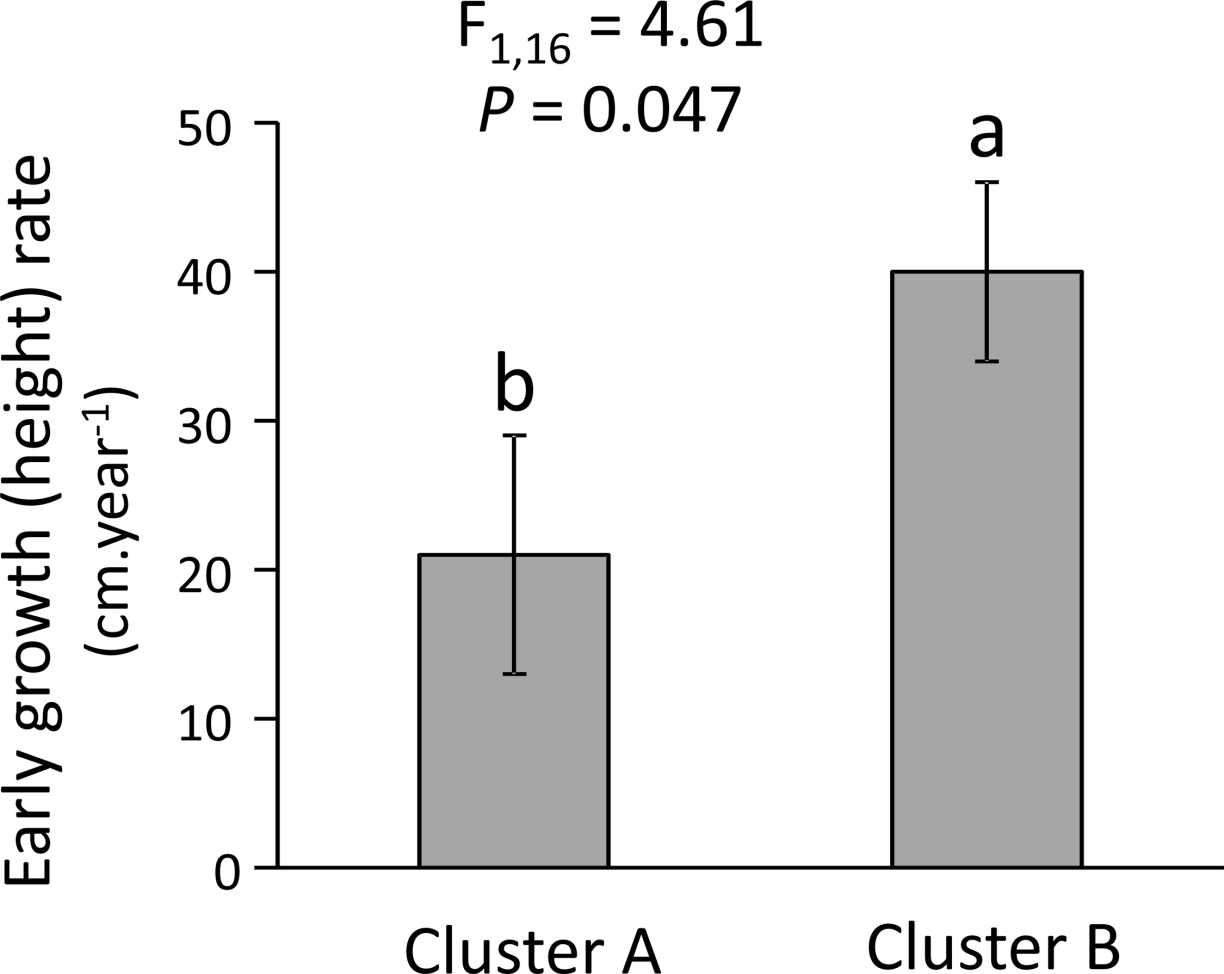
Differences in early plant growth (height) rate of 18 conifer species between the two defensive clusters. F and P-values of the effect of cluster after controlling for phylogeny using PGLS are shown. Bars are least square means ± s.e.m. (N = 9 species). Different letters indicate significant differences between defensive trait clusters.

Finally, we found that pines species in the two clusters significantly differed in their early height growth (Fig 3). Specifically, early growth rate of pine species included in Cluster B was nearly double that of pine species included in Cluster A (Fig 3).

## Discussion

Recent studies by our research group reported that both phylogeny and plant growth rate drive the expression of specific induced and constitutive chemical defense traits across pines species [28,31]. Here we go a step forward and show that plant growth rate, but not phylogenetic relatedness, determined the deployment of two divergent pine defense syndromes based on multiple constitutive and induced chemical defenses. In particular, slow-growing species prioritized the investment in high levels of constitutive resin in the needles and stem, whereas fast-growing species prioritized high inducibility of resin and phenolics in the needles by both JA and SA pathways. Although the concerted expression of both constitutive and induced defenses might be required for efficient plant defense against herbivores (i.e. combining immediate resistance after herbivory and reduction of metabolic costs, respectively), our study suggest that constraints on resource allocation probably have shaped the expression of both defensive strategies into divergent syndromes.

Our results showed that pine defense syndromes evolve in the direction proposed by the Resource Availability Hypothesis [16,27]. Specifically, slow-growing pine species adapted to harsh environments (e.g. high latitudes/altitudes associated with low temperatures and precipitations) that impose high costs of tissue replacement allocated more to constitutive defenses (despite they are energetically more costly to produce and maintain than those of induced strategies) [16,27,28,46]. In contrast, fast-growing pine species living in resource-rich habitats (e.g. low latitudes/altitudes associated with high temperatures and precipitations) have greater inducibility of their defenses, consistent with the constitutive-induced defense trade-offs observed in pine trees [28]. In accordance with our results, Fine et al. [15] documented that a trade-off between growth rate and defense investment caused plants living in white-sand and clay Amazonian forests to deploy divergent defensive strategies. Similarly, Kursar & Coley [6] reported that growth-defense trade-offs promoted divergent defensive strategies among tropical tree species.

Our clustering of pine species by phenotypic functional defensive traits was not congruent with phylogenetic relatedness among species, demonstrating that the integrated defense phenotype of pine trees is evolutionary labile [4,47,48]. Similar to our results, Agrawal & Fishbein [3] also observed that milkweed defense syndromes did not track phylogenetic history, suggesting that selection due to the biotic and abiotic environment drives these syndromes. Similarly, Haak et al. [18] found that local ecological context, rather than evolutionary history, shapes defense syndromes in wild tomatoes. Evolutionarily labile defense syndromes among pine species suggests that dynamic selective environments might act upon pine defensive traits. In this sense, it is important to note that the distribution range of our studied pine species span 17.38 million km^2^ (covering 11.7% of all the land on Earth), being widely distributed from sea level to high elevations and from very high latitudes to tropical areas [34]. Similarly, climatic conditions correlated with convergent defensive phenotypes of oaks [49], suggesting that local environments may be particularly important in shaping the defensive phenotypes of long-lived tree genera that cover large geographic areas in tropical and temperate regions.

Our approach is based on the macroevolutionary comparisons of traits among species sampled from single populations. This approach has been widely used in several herbaceous and woody species (e.g. [3, 10, 49, 50]) and is supported by the well-documented premise that among-species variation greatly exceeds within species variation (see [51]). However, it is also well-known that in conifers variation within species and populations within species in defense allocation is high (e.g. [52, 53]). Therefore, future work should also look at within-species variation in plant defense allocation for uncovering microevolutionary processes underlying macroecological patterns. Another fruitful avenue of future research would be to perform long-term field studies in order to address the ecological relevance of these defense syndromes at subsequent ontogenetic stages. In this sense, as both vegetative (e.g. rooting, growth) and defensive priorities change along the ontogeny it is likely possible that costs of chemical defense production will be transient along plant ontogeny (see [54]). Further studies should also include more defensive traits (e.g. tolerance related traits, physical defenses, and indirect defenses) and herbivory host records among geographical origins and species to determine if alternative mechanisms and defensive traits have been also important for the evolution of pine defense syndromes and under what conditions.

In conclusion, this study demonstrates that allocation traits determining the defensive phenotype in young pine trees are associated in two main groups or syndromes across the phylogeny of European and American pine species. Besides, our analyses provide strong evidence that constraints on resource allocation to growth, constitutive and induced defenses (but not phylogenetic relatedness between plant species) markedly determined the evolution of those defensive syndromes across pine tree species.

## Supporting Information

S1 Table Excel file including data used for this paper. (XLSX)
